# Comparison between CAD/CAM titanium mesh vs. conventional titanium mesh in bone regeneration: a systematic review and meta-analysis

**DOI:** 10.1186/s40729-025-00643-5

**Published:** 2025-08-22

**Authors:** Gian Maria Ragucci, Antonio Fernández Augè, Anna Tresserra Parra, Basel Elnayef, Federico Hernández-Alfaro

**Affiliations:** Department of Oral and Maxillofacial Surgery, Universitat Internacional de Catalunya, C. Josep Trueta, s/n, 08195 Sant Cugat del Vallès, Barcelona, Spain

**Keywords:** Titanium mesh, CAD/CAM titanium mesh, Guided bone regeneration, Vertical bone augmentation

## Abstract

**Background:**

Vertical bone defects remain a challenge in implant dentistry. Titanium mesh (TM) is widely used in guided bone regeneration due to its ability to stabilize grafts, but it requires intraoperative adaptation, increasing surgical time and the risk of complications like mesh exposure. Customized titanium mesh (CTM), designed using CAD/CAM or 3D printing, offers a precise fit and may reduce surgical risks. This systematic review and meta-analysis aims to compare CTM and TM in terms of bone gain and complication rates in vertical ridge augmentation procedures.

**Materials and methods:**

A systematic search was carried out in four electronic databases (PubMed, Cochrane Central, Web of Science, and Google Scholar) up to January 2025, with no time restrictions applied. Studies comparing customized titanium mesh (CTM) and conventional titanium mesh (TM) for vertical ridge augmentation were considered eligible if they included at least 10 patients and a minimum follow-up period of 6 months. The primary outcomes were vertical and horizontal bone gain, as well as membrane exposure. Meta-analyses and meta-regressions were performed using R software.

**Results:**

A total of 22 studies were included in the analysis (3 randomized controlled trials, 6 prospective studies, 12 retrospective studies, and 1 cohort study), comprising 608 patients and 1,318 implants. The mean vertical bone gain was 6.24 mm for the TM group and 5.14 mm for the CTM group, with no statistically significant difference between them (*P* = 0.628). In contrast, CTM achieved significantly greater horizontal bone gain (6.38 mm vs. 3.85 mm; *P* = 0.004). Membrane exposure occurred more frequently in the TM group (30.9%) than in the CTM group (20.3%), although the difference was not statistically significant (*P* = 0.721). Other complications, such as infections, were also more common in the TM group but did not show statistical significance.

**Conclusion:**

Within the limitations of the included studies, CTM appears to offer comparable bone gain to TM, with superior horizontal bone gain and a tendency to fewer complications. The results support the potential advantages of customized mesh in clinical practice. Further randomized trials with standardized protocols and long-term follow-up are recommended to confirm these findings.

## Graphical Abstract

**Figure:**
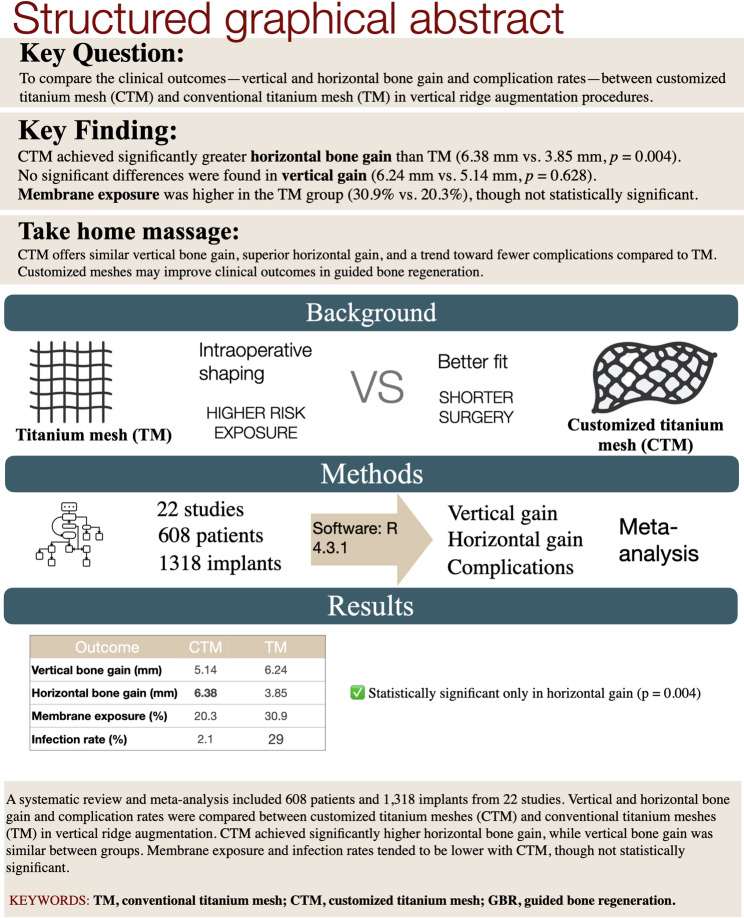
Overview of the study design and main outcomes comparing customized and conventional titanium mesh in guided bone regeneration. CTM showed greater horizontal bone gain and fewer complications, with similar vertical gain

## Introduction

Dental implant treatment has significantly evolved since its early stages, becoming a predictable and widely accepted technique [1]. It is now regarded as the gold standard for replacing lost teeth, with survival rates as high as 96.4% in long-term follow-ups [2]. Tooth extraction initiates a process of bone resorption, which, depending on individual and systemic factors, can lead to considerable changes in both the quantity and quality of the bone [3–5].

This bone resorption can result in clinical situations where it becomes impossible to rehabilitate patients with dental implants. To address these challenges, various surgical techniques have been developed, including the use of autogenous bone blocks, inlay blocks, guided bone regeneration, split crest/ridge expansion, nerve repositioning or lateralization, and sinus lift procedures in the posterior maxilla [6]. Despite their efficacy, these techniques may be associated with certain morbidities and complications. Such complications often arise from systemic or local factors related to the patient but are also directly influenced by the surgeon’s level of experience, the technical complexity of the procedure, and the correct application of the chosen technique based on the defect type and anatomical area, as well as the need to minimize surgical time [7, 8].

Titanium mesh (TM) is a guided bone regeneration technique that employs a titanium membrane alongside bone grafts, such as autologous bone and other substitutes like xenografts or allografts. Titanium is well-known for its excellent mechanical properties, which provide reliable stabilization for the graft [9]. In traditional methods, the titanium membrane required intrasurgical adaptation, which often necessitated large flaps. This could result in an imperfect fit, frequently leaving sharp edges. These sharp edges increased the risk of complications, such as graft exposure and infection, making the procedure more complex and time-consuming. Reported exposure rates in the literature range from 24 to 80% [7, 10–13].

To simplify this technique and eliminate the need for manual adaptation, a new generation of customized titanium meshes (CTM) has been developed using CAD/CAM technology. These meshes are tailored for each specific case and defect. The process involves analyzing the defect with cone beam computed tomography (CBCT), followed by the creation of a 3D titanium mesh through laser sintering, which ensures a precise and customized fit [14, 15]. This customized approach provides excellent fixation, securing the graft and promoting successful regeneration. The mesh design incorporates spaces that enable blood flow to the graft, supporting the formation of native bone. Additionally, pre-planned fixation screw placement ensures optimal positioning and avoids sensitive anatomical structures, thereby reducing intraoperative complications [16].

The use of a customized titanium mesh (CTM) allows for precise planning of future implant positioning, which results in shorter surgical times, reduced morbidity, and an anatomically accurate reconstruction of the defect. These advantages enhance vascularization during the regenerative process. This approach has demonstrated success in treating both horizontal and vertical bone defects [17–19].

Chiapasco et al., in a retrospective study using CTM, documented vertical and horizontal bone gains of 4.78 ± 1.88 mm and 6.35 ± 2.10 mm, respectively, with a follow-up of 10.6 ± 6.5 months and a survival rate of 100% [17]. Similarly, Sagheb et al. reported a 100% survival rate in 44 implants placed with CTM and a follow-up of 12 ± 6 months [18].

The objective of this systematic review is to analyze the clinical and radiological outcomes—including vertical and horizontal bone gain—as well as the potential complications and challenges associated with the use of customized versus conventional titanium mesh in bone augmentation procedures.

## Materials and methods

### Search strategy

Four electronic databases- Cochrane Central, Google Scholar, PubMed and Web of Science - were used to identify relevant publications in English without time restrictions. The search was conducted up to January 2025 by two independent reviewers (G.M.R and A.T.P) to address the proposed PICO (Patient, Intervention, Comparison, and Outcome) questions: In patients over 18 years of age, what are the differences in terms of bone gain, surgical complications, and implant survival between using TM and CTM for vertical bone regeneration?

Search terms included: “guided bone regeneration,” “vertical guided bone regeneration,” “titanium mesh,” “CAD/CAM titanium mesh,” “conventional titanium mesh,” and “computer-aided manufacturing titanium mesh.”

Additionally, a manual review of references from the included studies was performed. The selected studies (January 2000–January 2025) were published in leading dental journals, such as the *Journal of Oral Maxillofacial Implants*,* Implant Dentistry*,* European Journal of Oral Implantology*,* Journal of Oral Implantology*,* International Journal of Oral and Maxillofacial Surgery*,* Journal of Oral and Maxillofacial Surgery*,* Journal of Dental Research*,* International Journal of Prosthodontics*,* Journal of Prosthetic Dentistry*,* Journal of Clinical Periodontology*,* Journal of Periodontology*,* The International Journal of Periodontics and Restorative Dentistry*, and *Clinical Implant Dentistry & Related Research.*

### Inclusion and exclusion criteria

To be eligible for inclusion, studies had to report on human prospective or retrospective trials examining the outcomes of guided bone regeneration procedures using either TM or CTM. Only studies with a minimum follow-up period of six months and at least ten participants per group were included in the qualitative assessment. Studies were excluded if they were case reports, case series, in vivo or in vitro experiments, systematic reviews, or had fewer than six months of follow-up or fewer than ten participants per group.

### Outcomes

The primary outcomes assessed in this systematic review and meta-analysis were as follows:

1. Vertical bone gain.
2. Horizontal bone gain.
3. Titanium mesh exposure.

### Study selection

Two independent reviewers (G.M.R and A.T.P) screened all titles and abstracts to identify studies for further evaluations. Full-text articles of potentially eligible studies were retrieved and independently reviewed. Any disagreements were resolved through consultation with a third reviewer (F.H.A).

### Quality assessment

The quality of the included randomized controlled trials (RCTs) was assessed using criteria adapted from the Cochrane Center’s randomized clinical trial checklist and the CONSORT (Consolidated Standards of Reporting Trials) guidelines. The assessment included the following parameters:

1. Randomization sequence generation.
2. Allocation concealment methods.
3. Blinding of the examiner.
4. Management of incomplete outcome data.
5. Avoidance of selective outcome reporting.

Each study was independently reviewed by G.M.R and A.T.P to ensure compliance with these standards.

### Statistical analysis

The meta-analysis was performed using R software version 4.3.1 (R Core Team; R Foundation for Statistical Computing; Vienna, Austria). A random-effects model was applied to account for variability among studies, with a restricted maximum likelihood estimator used to assess heterogeneity. Weighted means (WM) and 95% confidence intervals (CI) were calculated for vertical and horizontal bone gain, titanium mesh exposure rates, and other complications.

Meta-regressions were conducted to evaluate the influence of the technique (conventional vs. CAD/CAM titanium meshes) as a moderator variable. Effect sizes and confidence intervals were visually represented in Forest plots, while funnel plots and Egger’s test were used to assess publication bias. Heterogeneity was quantified using the I² statistic, and Cochran’s Q test was employed to evaluate its significance.

Sensitivity analyses were performed by excluding studies with high heterogeneity to ensure the robustness of the results. A significance level of α = 0.05 was applied for all statistical tests, providing a reliable and comprehensive evaluation of the data.

### Risk of bias assessment

The Cochrane Risk of Bias Tool version 2 (RoB-2) was applied to randomized controlled trials, evaluating the following domains: randomization process, deviations from intended interventions, missing outcome data, outcome measurements, and selection of reported results. For non-randomized studies, including prospective and retrospective designs, the Newcastle-Ottawa Scale (NOS) was used to assess study quality across three domains: selection, comparability, and outcome. All assessments were independently performed by two reviewers (A.F.A. and G.M.R.), and any discrepancies were resolved through discussion with a third reviewer (F.H.A.)

This systematic review was registered in the International Prospective Register of Systematic Reviews (PROSPERO) under the number CRD420251051175.

## Results

**Fig. 1:**
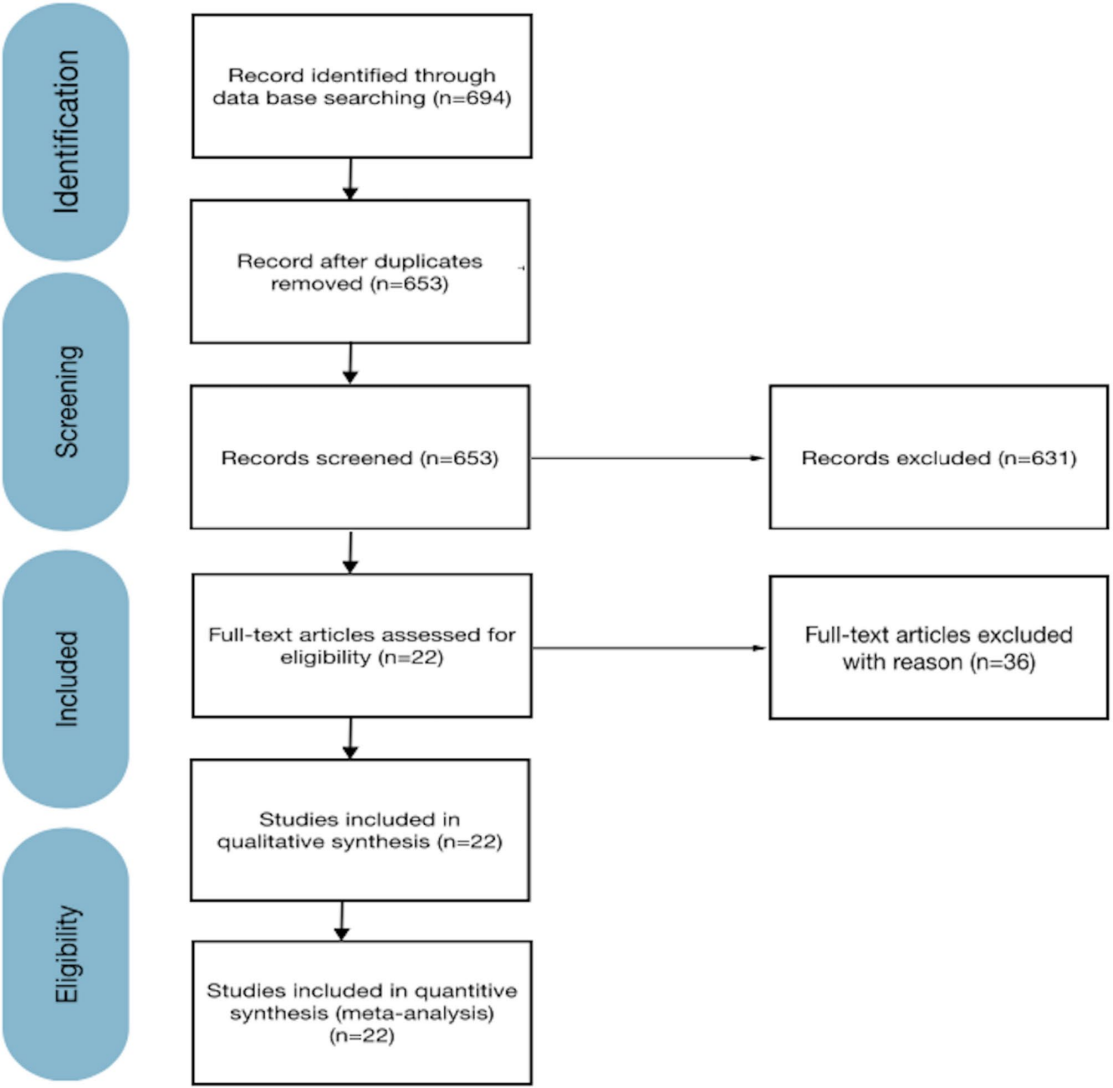
PRISMA flow diagram illustrating the selection process of included studies

The initial search identified 695 titles, of which 56 were identified as potentially relevant after the initial screening phase. Following a second screening, full-text publications were obtained and reviewed, resulting in 22 studies that met the inclusion criteria. These included three randomized controlled trials, one cohort study, 12 retrospective studies, and six prospective studies. Articles were excluded for reasons such as lack of data on vertical bone gain, sample sizes smaller than 10 participants, follow-up periods shorter than six months, or missing follow-up timing information. All included studies had an average follow-up of at least six months, with four studies reporting follow-up durations exceeding 18 months (Corinaldesi et al. [19], El Chaar et al. [20], Louis et al. [21], and Miyamoto et al. [22]). The longest follow-up recorded was 66.6 months in the study of Miyamoto et al. In total, 608 patients and 1.318 implants were analyzed. The study selection process is illustrated in Fig. 1.

Table 1 provides a detailed summary of the included studies, including design, number of implants and patients, mesh type, follow-up period, and study results.

**Table 1:** Summary of the main characteristics and outcomes of the included studies

Author	Year	Type of study	Nº of patients	Nº of implants	Type of mesh	Bone Graft	Bone gain Vertical (mm)	Bone gain horizontal (mm)	Membrane exposure (%)	Other complications (%)	Follow-up (months)
Chiapasco et al. [17]	2021	RS	41	106	CAD/CAM	MIX: Auto + DBBM	4.78 ± 1.88	6.35 ± 2.10	20.7	2.5	10.6 ± 6.5
Corinadelsi et al. [19]	2009	RS	24	56	Conventional	Auto	5.45 ± 1.16	NA	14. 8	NA	39–96
Cucchi et al. [28]	2017	RCT	20	108	Conventional	MIX: Auto + ALO	4.1 ± 1.0	NA	21.1	15.8	6–9
Dellavia et al. [26]	2021	CS	20	48	CAD/CAM	MIX: Auto + DBBM	5.20 ± 1.76	6.8 ± 2.51	15	NA	9
El Chaar et al. [20]	2019	RS	39	63	Conventional	Allograft	6.37	6.42	46.15	10.25	18–48
Gomes et al. [31]	2016	RS	25	40	Conventional	DBBM	NA	3.67 ± 0.89	24	48	12
Hartmann et al. [33]	2020	RS	55	98	CAD/CAM	MIX: Auto + DBBM	NA	NA	25	NA	6.53 ± 2.7
Her et al. [10]	2012	RS	26	69	Conventional	DBBM	NA	NA	26	NA	6–24
Jung et al. [34]	2014	PS	10	12	CAD/CAM	MIX: Auto + ALO	NA	NA	3	NA	12
Lizio et al. [13]	2016	PS	24	88	Conventional	MIX: Auto + DBBM	NA	NA	58.82	11.76	6–12
Louis et al. [21]	2008	RS	44	174	Conventional	MIX: Auto + DBBM	13.5 ± 1.7	NA	52.3	NA	3-6-9
Miyamoto et al. [22]	2012	RS	27	87	Conventional	Auto	5.4 ± 3.4	3.7 ± 2.0	40.7	66.66	47.5
Poli et al. [32]	2014	RS	13	20	Conventional	MIX: Auto + DBBM	NA	NA	7.69	NA	88
Rocuzzo et al. [24]	2007	PS	12	NA	Conventional	Autogenous Block	5.7 ± 1.5	3.4	33.3	NA	4–6
Sagheb et al. [18]	2017	PS	17	44	CAD/CAM	MIX: Auto + DBBM	6.5 ± 1.7	5.5 ± 1.9	33	NA	6–12
Torres et al. [25]	2010	RCT	30	97	Conventional	Anorganic bovine Bone (ABB)	3.3 ± 0.2	3.9 ± 0.2	20	NA	24
Nan et al. [27]	2023	RS	59	80	CAD/CAM	MIX: Auto + DBBM	5.01 ± 2.83	5.22 ± 3.19	32.8	NA	12
Li et al. [30]	2023	RS	36	57	CAD/CAM	MIX: Auto + DBBM	2.38 ± 2.69	5.37 ± 2.01	16.67	NA	6–8
Cucchi et al. [12]	2021	RCT	30	71	CAD/CAM	MIX: Auto + DBBM	5.55 ± 2.38	NA	23.3	6.6	6
Pellegrino et al. [28]	2024	PS	16	NA	CAD/CAM	MIX: Auto + DBBM	5,89 ± 0.45	9.52 ± 0.65	12.5	NA	8
Chen et al. [16]	2023	RS	30	NA	CAD/CAM	MIX: Auto + DBBM	5.02 ± 1.42	NA	30	0	6
Hernández-Alfaro et al. [29]	2024	PS	10	NA	CAD/CAM	MIX: Auto + DBBM	5.87 ± 1.6	5.64 ± 2.1	20	0	6

**Abbreviations**: RCT, randomized controlled trial; RS, retrospective study; PS, prospective study; CS, case series; Auto, autologous bone; DBBM, deproteinized bovine bone mineral; ALO, allograft; ABB, anorganic bovine bone; CAD/CAM, computer-aided design/computer-aided manufacturing; NA, not available

### Vertical bone gain

**Fig. 2:**
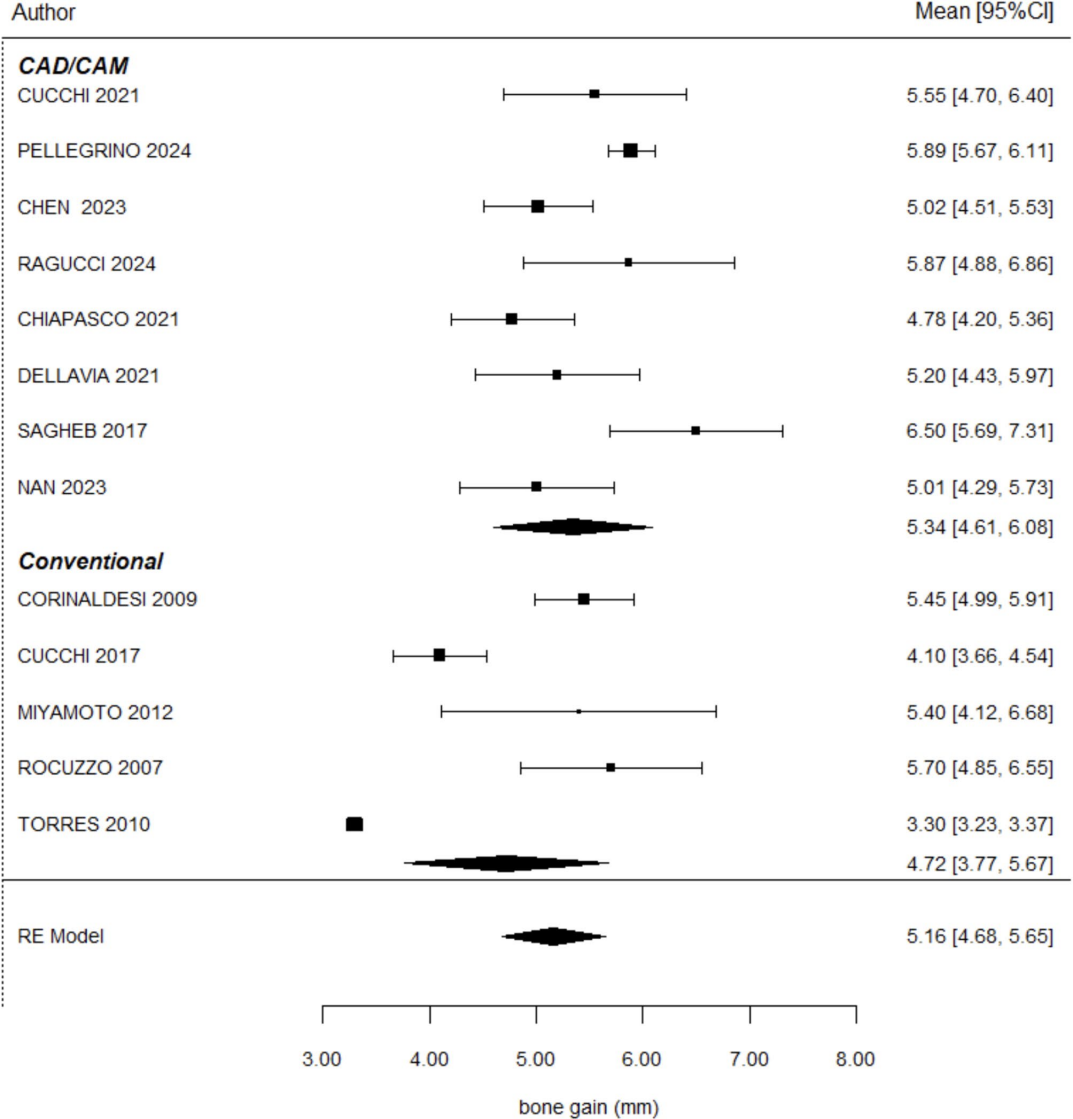
Forest plot comparing vertical bone gain between conventional titanium meshes (TM) and customized titanium meshes (CTM). No statistically significant difference was found between groups (*P* = 0.628). Confidence intervals and heterogeneity indices are reported

Vertical bone gain was analyzed in 15 articles with CBCT [12, 16–19, 21–30]. The mean vertical bone gain recorded with TM was 6.24 ± 1.51 mm, while for CTM, the mean value was 5.14 ± 0.38 mm. No significant differences were observed between the two groups (*P* = 0.628). These results are illustrated in Fig. 2, which presents the forest plot comparing vertical bone gain between TM and CTM. The studies by Louis et al. [21] and Li et al. [30] were excluded from the meta-analysis on vertical bone gain due to their significant contribution to heterogeneity and outlier data. Louis presented markedly inconsistent values compared to other studies, while Li reported exceptionally low outcomes. Similar outcomes regarding vertical bone gain using CAD/CAM customized titanium meshes were also reported in other non-comparative studies, including those by Hernández-Alfaro et al. [29], Nan et al. [27], and Dellavia et al. [26], with reported gains of 5.87 mm, 5.2 mm, and 5.1 mm, respectively.

### Horizontal bone gain

Regarding horizontal gain, 10 articles reported results [17, 18, 22, 25, 26, 27, 28, 29, 30, 31]. The mean horizontal gain recorded was 3.85 ± 0.08 mm for TM and 6.38 ± 0.60 mm for CTM, respectively.

**Fig. 3:**
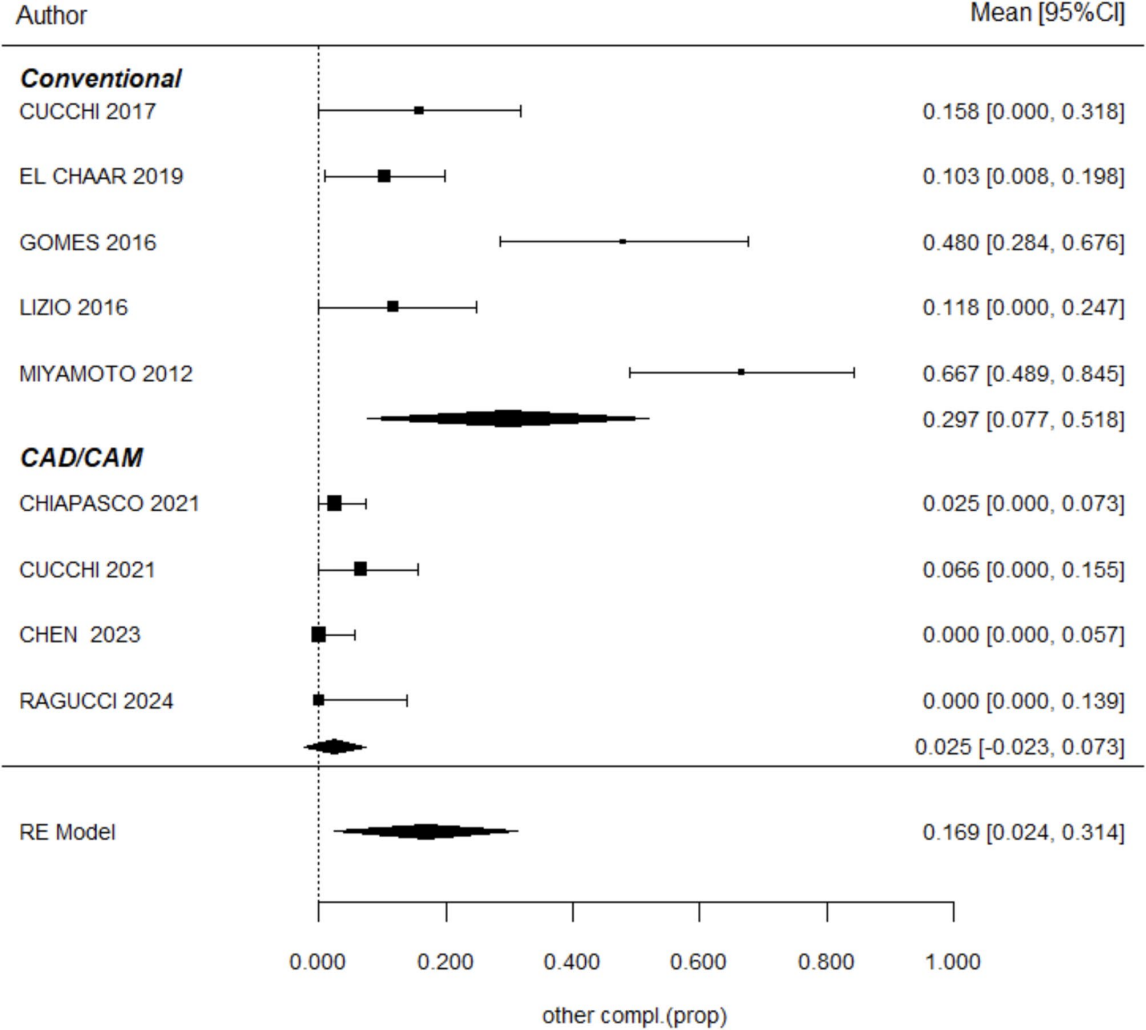
Forest plot comparing horizontal bone gain between CTM and TM. CTM showed a significantly greater gain of 1.61 mm (*P* = 0.004), with 55.5% of the heterogeneity explained by the technique used. Confidence intervals and heterogeneity indices are provided

Therefore, horizontal augmentation with CTM was, on average, 1.61 mm greater than with TM mesh. This difference in horizontal gain between the groups was statistically significant (*P* < 004), as illustrated in Fig. 3, which displays the forest plot comparing horizontal bone gain between the two techniques.

### Membrane exposure

**Fig. 4:**
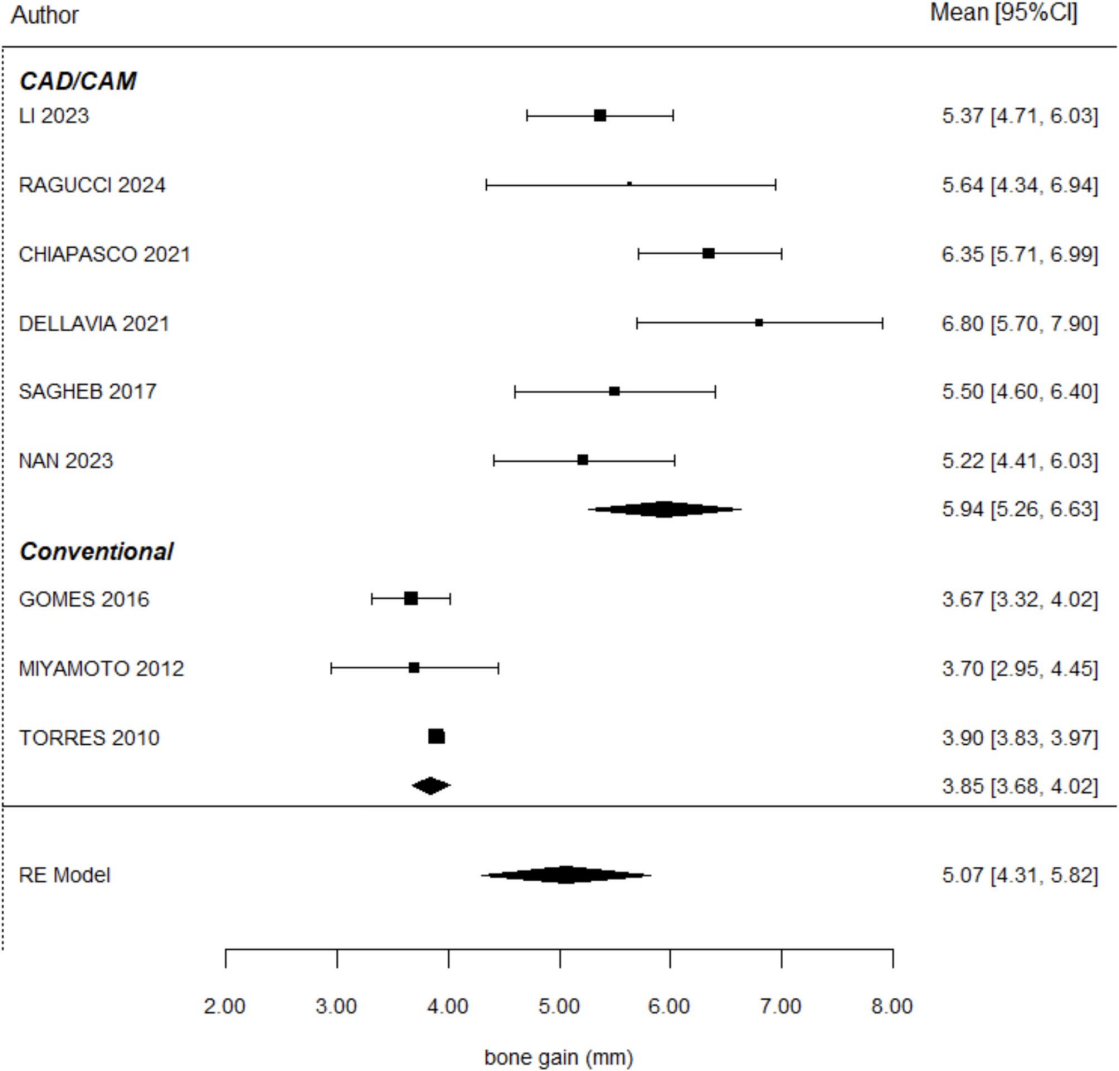
Meta-regression results comparing membrane exposure rates between conventional and CAD/CAM titanium meshes. CAD/CAM meshes showed a 2.4% lower exposure rate than conventional meshes; however, the difference was not statistically significant (*P* = 0.721). Confidence intervals, z-scores, and heterogeneity indices are reported

Membrane exposure complications were analyzed across 22 studies by clinical assessment, resulting in a mean exposure rate of 26.8% [10, 12, 13, 16–34]. For TM, the membrane exposure rate was 30.9% ± 5%, while CTM showed a rate of 20.3% ± 3.1%. No significant differences were observed between the groups (*P* = 0.721). These results are summarized in Fig. 4, which presents the forest plot comparing membrane exposure rates between TM and CTM.

### Other complications

**Fig. 5:**
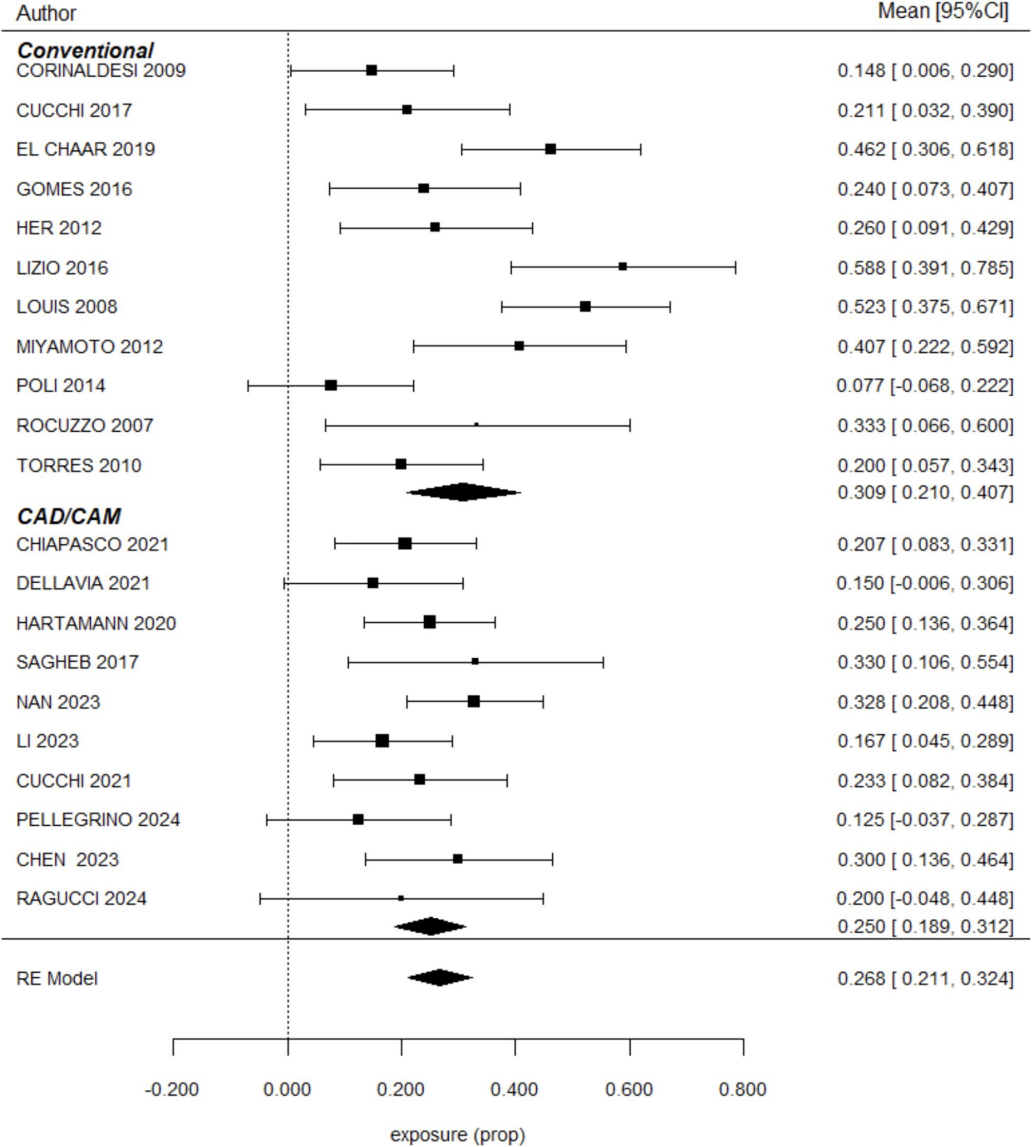
Meta-regression results evaluating the incidence of other complications between the two groups. CAD/CAM titanium meshes demonstrated a 16.4% lower complication rate compared to conventional meshes, though this difference was not statistically significant (*P* = 0.491). Confidence intervals, z-scores, and heterogeneity indices are presented for detailed interpretation

**Fig. 6:**
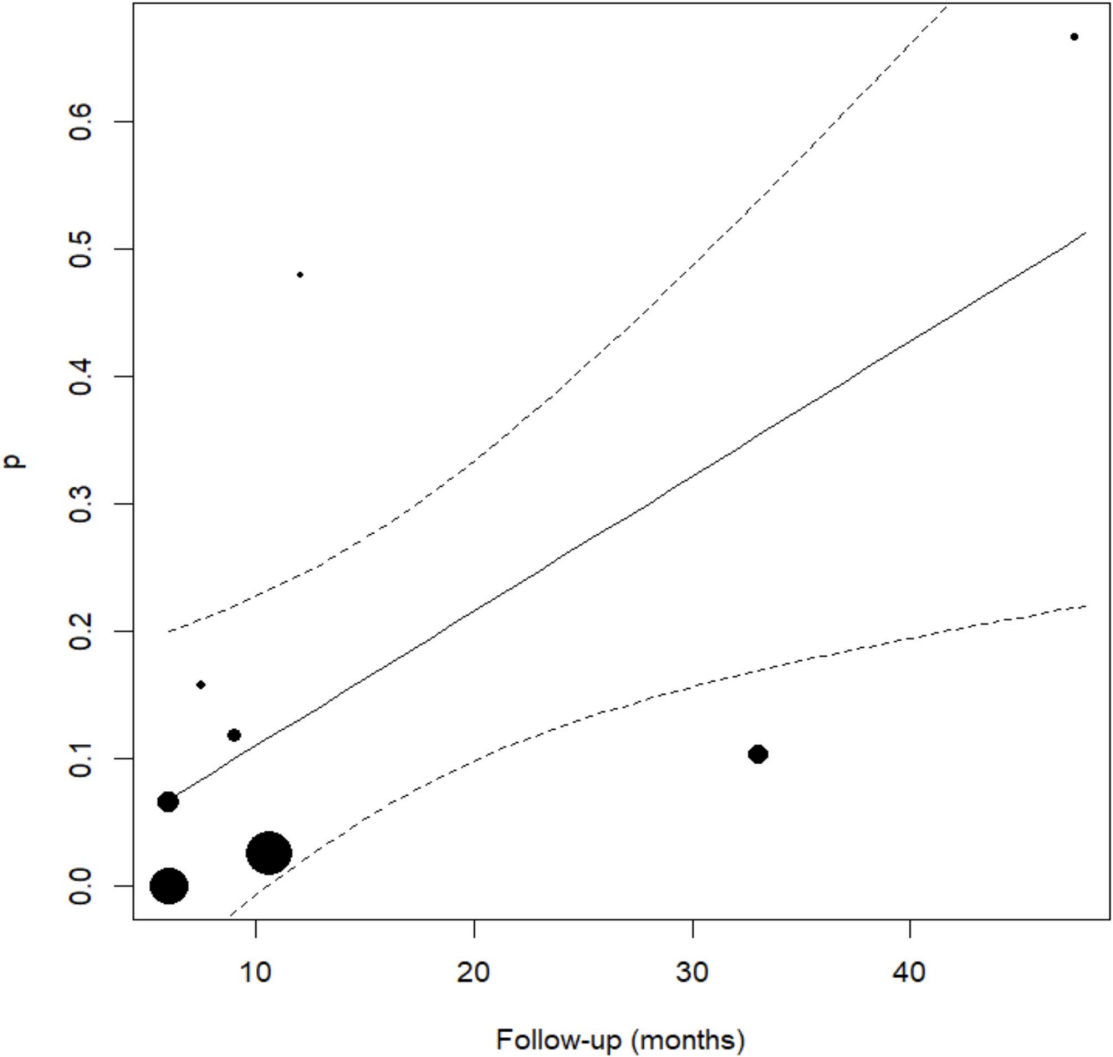
Scatterplot illustrating the association between follow-up duration and the rate of other complications across studies using conventional and CAD/CAM titanium meshes. A trend is observed whereby each additional month of follow-up correlates with a ~ 1% increase in complication rate (*P* = 0.021). The regression line reflects this association

Infection was identified as the most frequent complication, outcomes were reported in 9 studies [12, 13, 16, 17, 20, 22, 23, 29, 31]. The complication rate in the TM group was 29% ± 11.3%, compared to 2.1% ± 1.7% in the CTM group (*P* = 0.558). Although the difference between the techniques was not statistically significant, there appears to be a tendency for a higher rate of infections in the TM group. These findings are summarized in Fig. 5. In addition, Fig. 6 illustrates the meta-regression analysis, showing that follow-up time is a significant factor influencing complication rates (*P* = 0.021). Specifically, each additional month of follow-up increases the rate of complications by approximately 1%.

### Risk of bias results

The RoB-2 tool was applied to three randomized controlled trials. Torres et al. [27] was rated as having a low risk of bias, while the other two studies [12, 23] were judged to present some concerns, mainly due to incomplete outcome data and insufficient reporting of the randomization process.

The remaining 22 studies were assessed using the Newcastle-Ottawa Scale (NOS), with scores ranging from 6 to 9 stars. Seven studies were classified as high quality (≥ 7 stars), while the rest were considered of moderate quality. The most frequent limitations were the absence of control groups and the lack of multivariable adjustments. A detailed summary of the risk of bias assessments is provided in Tables 2 and 3.

**Table 2:** Domain-based risk of bias assessment (RoB-2) for randomized controlled trials included in the review

	D1	D2	D3	D4	D5	Overall
Cuchi et al. 2017	-	+	-	+	+	-
Cochi et al. 2021	+	+	-	+	+	-
Torres et al. 2010	+	+	+	+	+	+

D1: Bias arising from the randomization process; D2: Bias due to deviation from intended intervention; D3: Bias due to missing outcome data; D4: Bias in measurement of the outcome; D5: Bias in selection of the reported results; “+” Low risk; “-” some concerns

**Table 3:** Risk of bias assessment of non-randomized studies using the Newcastle-Ottawa scale (NOS)

	Number of awarded stars in each domain			
	Selection	Comparability	Outcome	Study Quality
Chapiasco et al. 2021	3	1	3	High
Corinadelsi et al. 2009	3	1	3	High
Li et al. 2023	4	1	3	High
Pellegrino et al. 2024	3	0	3	Moderate
Rocuzzo et al. 2007	4	1	3	High
Sagheb et al. 2017	3	0	3	Moderate
Lizio et al. 2016	3	0	3	Moderate
Dellavia et al. 2021	3	0	3	Moderate
El Chaar et al. 2019	4	1	3	High
Gomes et al. 2016	3	0	3	Moderate
Hartmann et al. 2020	3	0	3	Moderate
Her et al. 2012	3	0	3	Moderate
Jung et al. 2014	3	0	3	Moderate
Poli et al. 2014	3	0	3	Moderate
Ragucci et al. 2024	3	0	3	Moderate
Miyamoto et al. 2012	3	0	3	Moderate
Louis et al. 2008	3	0	3	Moderate
Nan et al. 2023	3	0	3	Moderate
Chen et al. 2023	4	1	3	High

NOS: maximum score = 9 points. Domains: Selection (max. 4), Comparability (max. 2), Outcome (max. 3). Higher scores indicate better methodological quality

## Discussion

In the current literature, there are still few studies that directly compare the outcomes of conventional titanium mesh (TM) and customized titanium mesh (CTM) in guided bone regeneration procedures. One of the most recent systematic reviews on this topic was conducted by Zhou et al., who performed a meta-analysis focusing mainly on the exposure rates between the two types of mesh. Their results showed a lower exposure rate for CTM (31%) compared to TM (51%). However, this review presents certain limitations. Most of the included studies were retrospective, with only one randomized controlled trial, and the analysis was centred only on exposure rates, without taking into account other important clinical outcomes such as vertical or horizontal bone gain, implant success or postoperative complications. Therefore a more comprehensive review is still necessary, besides exposure rates, also looks at other key clinical outcomes like bone gain and implant success, which are crucial for everyday clinical practice [35].

The primary objective of the present study was to assess and analyze the outcomes associated with both TM and CTM by reviewing the available literature and comparing their clinical performance. The findings indicated that the CTM technique demonstrated greater horizontal bone gain in comparison to the TM group. Regarding vertical bone gain, no statistically significant differences were found between the two groups. Although there was a noticeable tendency for fewer complications in the CTM group, this difference did not reach statistical significance, both in terms of membrane exposure rates and other complications, such as infections and dehiscences.

In this study, the mean vertical bone gain across groups was 5.65 mm, and the horizontal gain was 5.20 mm. These values seem to be higher than those reported by Sabri et al., who recently published a systematic review and meta-analysis focused on bone gain outcomes with titanium mesh. They found a mean vertical gain of 3.36 mm and a horizontal gain of 3.26 mm, although there was some variability among the included studies [36]. Unlike Zhou et al., who mainly focused on exposure rates, Sabri’s study looked at bone gain and also compared titanium mesh to other membranes like collagen. Still, it didn’t include a direct comparison between conventional and customized titanium meshes. In fact, only two of the 22 studies in their review evaluated results with customized titanium mesh, so the available evidence on that is still quite limited [35, 36].

In terms of vertical bone gain, the mean gain observed in the CTM group was 5.14 ± 0.38 mm. On the other hand, in the TM group, the mean vertical bone gain was 6.24 ± 1.51 mm. However, this value exhibited considerable variability across the included studies. For instance, the study conducted by Louis et al. reported a notably higher mean vertical gain of 13.5 ± 1.7 mm, while Torres et al. observed a considerably lower gain of only 3.3 ± 0.2 mm [21, 25]. Such discrepancies could likely be attributed to multiple factors, including differences in defect types (e.g., wide defects vs. narrow defects), measurement techniques (e.g., radiographic vs. clinical measurements) or surgical protocols (e.g., use of fixation devices, barrier membranes, or simultaneous implant placement). These variations highlight the complexity involved in interpreting vertical bone gain outcomes and emphasize the need for standardized protocols in future studies.

Regarding horizontal bone gain, the results showed a mean gain of 6.38 ± 0.60 mm in the CTM group. These results are consistent with the findings of Chiapiasco et al., who reported a mean horizontal gain of 6.35 ± 2.10 mm, suggesting comparable outcomes between both investigations [17]. Conversely, Pellegrino et al. observed a significantly greater mean horizontal gain of 9.3 mm [30]. This substantial discrepancy could be attributed to differences in defect size (larger defects may require greater regenerative volumes), graft volume (a higher volume may promote greater horizontal bone gain) or surgical techniques (variations in flap design, graft fixation, or membrane application) In the TM group, the mean horizontal gain was 3.85 ± 0.08 mm, a result that differs notably from the findings reported by El Chaar et al. in that retrospective study involving 39 patients, a higher mean horizontal gain of 6.42 mm was reported. This inconsistency may reflect differences in defect characteristics, patient selection, or surgical expertise [20]. The superior horizontal gain observed in the CTM group may be attributed to the customized mesh design, which allows for improved adaptation to the unique morphology of each defect, enhanced fixation, and superior stability of both the mesh and the graft material. Furthermore, titanium mesh produced through the 3D printing selective laser melting method exhibits robust mechanical properties and a smooth surface [35, 37].

It should be acknowledged that the observed difference in horizontal bone gain between CTM and TM may be influenced by anatomical heterogeneity among the included studies. Specifically, several studies involving CTM focused on posterior defects, particularly in the molar region, where larger horizontal augmentation is often required. In contrast, TM was frequently applied in anterior sites, where the morphology of the defect may limit the achievable horizontal gain. This distribution bias could partially explain the significant difference in horizontal bone gain observed in favor of CTM. Unfortunately, most of the included studies did not provide a detailed stratification of results according to defect location, limiting the possibility of subgroup analysis. Future studies should control for anatomical site to more accurately assess the impact of mesh type on bone gain.

The reported overall exposure rate was 25.3%, with 30.9% in the TM group and 20.3% in the CTM group. Although the CTM group exhibited a lower incidence of membrane exposure, this difference was not statistically significant. These findings align with those reported by Gu et al., who described a mean exposure rate of 22.7%, with 19.9% in the TM group and 25.2% in the CTM group [38]. On the contrary, Zhou et al. reported significantly higher exposure rates, with an overall exposure rate of 43%, including 51% in the TM group and 31% in the CTM group [35]. This notable difference may be attributed to disparities in sample sizes (608 patients in the present study vs. 247 patients in Zhou’s study) or greater heterogeneity in the study populations and surgical protocols.

Beyond membrane exposure, other complications such as dehiscence, partial or total graft loss, necrosis and abscess formation were observed. These complications were recorded in 29.8% ± 11.3 of cases in the TM group and 2.1% ± 1.7 in the CTM group. While this difference appears substantial, it was not statistically significant. Consistent with the overall exposure rate found in the conventional group (30.9%), individual studies such as those by Gomes et al. [31] and Poli et al. [32] also reported relatively high rates of membrane exposure, reinforcing the clinical relevance of this complication in non-customized protocols. In line with the general exposure rate found in the CAD/CAM group (20.3%), Jung et al. [34] reported titanium mesh exposure in 2 out of 10 patients (20%), despite the use of preformed, customized meshes. The authors emphasized that although minor exposures occurred, bone regeneration and implant stability were not compromised.

In a separate analysis, Urban et al. conducted a systematic review and meta-analysis comparing the effectiveness of different VRA techniques and their associated complications [39]. Their results showed that osteogenic distraction achieved the greatest vertical bone gain, with an average of 8.04 mm, followed by the inlay block technique, which had a mean gain of 3.46 mm. Urban’s study also examined the GBR technique, incorporating both non-resorbable and resorbable membranes, as well as titanium mesh. The mean vertical bone gain achieved with GBR was 4.18 mm. While osteogenic distraction provided the highest vertical gain, it also showed the highest complication rate at 47.3%, nearly double the rate observed for titanium mesh in the present study. Both Urban et al. and Alotaibi et al. reported comparable results, with customized titanium mesh achieving a mean vertical gain of 5.2 mm [7]. Both studies concluded that while osteogenic distraction achieved the greatest bone gain, it was also linked to the highest complication rate. Conversely, the GBR technique emerged as the most balanced approach, providing satisfactory bone gain with a lower risk of complications. Another study reported a premature exposure rate of 25.7% for d-PTFE membranes [40]. 

The exclusion criteria for this study required a minimum follow-up period of six months and a sample size of at least 10 patients, which may have excluded relevant studies. Furthermore, there was no standardization in the measurement methods, and the included studies provided limited data on the shape and size of the treated defects. These factors may have introduced bias in the results. Considering these limitations is crucial when making clinical decisions. Future studies should focus on conducting well-designed randomized controlled trials with larger sample sizes, extended follow-up periods, and standardized defect characteristics to further validate these findings.

The results of this review suggest that customized titanium meshes (CTM) may offer clinical advantages over conventional meshes, particularly regarding surgical handling and horizontal bone gain. The individual adaptation of the mesh facilitates easier contouring and soft tissue closure, potentially reducing the risk of complications such as infection or dehiscence. Although no significant differences were found in vertical bone gain or membrane exposure, the greater predictability in horizontal outcomes may be especially beneficial in cases requiring precise contour or volume. These findings may help guide clinicians in selecting the most appropriate technique for vertical ridge augmentation procedures.

## Conclusions

Within the limitations of this study, the use of customized titanium mesh (CTM) appears to be an effective technique for bone augmentation. While no significant differences were observed compared to conventional titanium mesh in terms of vertical bone gain and complications, customized titanium mesh demonstrated superior outcomes in horizontal bone regeneration.

The results should be interpreted with caution, as the included studies may have been influenced by uncontrolled confounding factors. To gain a clearer understanding of their potential impact, additional randomized controlled trials with extended follow-up periods are recommended.

## Data Availability

No datasets were generated or analysed during the current study.

## Abbreviations

T M: Titanium Mesh
CTM: Customized Titanium Mesh
CAD/CAM: Computer-Aided Design / Computer-Aided Manufacturing
CBCT: Cone Beam Computed Tomography
VRA: Vertical Ridge Augmentation
GBR: Guided Bone Regeneration
RCT: Randomized Controlled Trial
NOS: Newcastle-Ottawa Scale
RoB-2: Risk of Bias Tool version 2 (Cochrane)
WM: Weighted Mean
CI: Confidence Interval
PRISMA: Preferred Reporting Items for Systematic Reviews and Meta-Analyses
PICO: Patient, Intervention, Comparison, Outcome
